# Cardiovascular magnetic resonance parameters associated with early transplant-free survival in children with small left hearts following conversion from a univentricular to biventricular circulation

**DOI:** 10.1186/s12968-014-0073-1

**Published:** 2014-10-07

**Authors:** Puja Banka, Barbara Schaetzle, Rukmini Komarlu, Sitaram Emani, Tal Geva, Andrew J Powell

**Affiliations:** Department of Cardiology, Boston Children’s Hospital, 300 Longwood Ave, Boston, MA 02115 USA; Department of Cardiac Surgery, Boston Children’s Hospital, 300 Longwood Ave, Boston, MA 02115 USA; Department of Pediatrics, Harvard Medical School, Boston, MA USA; Department of Surgery, Harvard Medical School, Boston, MA USA; Current address: Kantonsspital Winterthur, Winterthur, Switzerland; Current address: Department of Pediatric Cardiology, Cleveland Clinic, Cleveland, OH USA

**Keywords:** Hypoplastic left heart syndrome, Atrioventricular canal, Cardiovascular magnetic resonance, Pediatrics, Congenital heart disease, Surgery, Biventricular repair

## Abstract

**Background:**

We sought to identify cardiovascular magnetic resonance (CMR) parameters associated with successful univentricular to biventricular conversion in patients with small left hearts.

**Methods:**

Patients with small left heart structures and a univentricular circulation who underwent CMR prior to biventricular conversion were retrospectively identified and divided into 2 anatomic groups: 1) borderline hypoplastic left heart structures (BHLHS), and 2) right-dominant atrioventricular canal (RDAVC). The primary outcome variable was transplant-free survival with a biventricular circulation.

**Results:**

In the BHLHS group (n = 22), 16 patients (73%) survived with a biventricular circulation over a median follow-up of 40 months (4–84). Survival was associated with a larger CMR left ventricular (LV) end-diastolic volume (EDV) (p = 0.001), higher LV-to-right ventricle (RV) stroke volume ratio (p < 0.001), and higher mitral-to-tricuspid inflow ratio (p = 0.04). For predicting biventricular survival, the addition of CMR threshold values to echocardiographic LV EDV improved sensitivity from 75% to 93% while maintaining specificity at 100%. In the RDAVC group (n = 10), 9 patients (90%) survived with a biventricular circulation over a median follow-up of 29 months (3–51). The minimum CMR values were a LV EDV of 22 ml/m^2^ and a LV-to-RV stroke volume ratio of 0.19.

**Conclusions:**

In BHLHS patients, a larger LV EDV, LV-to-RV stroke volume ratio, and mitral-to-tricuspid inflow ratio were associated with successful biventricular conversion. The addition of CMR parameters to echocardiographic measurements improved the sensitivity for predicting successful conversion. In RDAVC patients, the high success rate precluded discriminant analysis, but a range of CMR parameters permitting biventricular conversion were identified.

## Background

Children born with cardiac malformations can have a range of left ventricular underdevelopment. At one end of the spectrum, patients may have severe ventricular hypoplasia and/or valve atresia rendering a biventricular circulation with the pulmonary and systemic ventricles in series impossible. At the other end are mild degrees of valve stenosis and/or ventricular hypoplasia that are compatible with a biventricular circulation. Between these extremes are patients in whom the decision whether to pursue univentricular palliation or biventricular repair is both challenging and risky.

This clinical dilemma most commonly arises in the context of 2 anatomic diagnoses: 1) patients who have various combinations of left-sided valvar hypoplasia and stenosis (mitral and/or aortic), left ventricular (LV) hypoplasia, and aortic coarctation (borderline hypoplastic left heart structures, BHLHS); and 2) those who have right-dominant atrioventricular canal defects (RDAVC) with variable degrees of right ventricular (RV) dominance, abnormalities of the left ventricular papillary muscle and chordal architecture, and left ventricular hypoplasia. In both situations, the decision whether to proceed with a biventricular circulation management strategy has profound implications. Pursuit of univentricular palliation commits a patient to the lifelong consequences of a Fontan circulation with its associated risks of mortality, ventricular and valvar dysfunction, heart failure, arrhythmia, protein losing enteropathy, and liver dysfunction [1-5]. However, inappropriate pursuit of a biventricular repair carries with it the threat of perioperative mortality, LV systolic and diastolic dysfunction, valvar dysfunction requiring frequent repairs or replacement, and pulmonary hypertension [6].

Despite the important ramifications of the decision to pursue a univentricular versus biventricular circulation, current management algorithms, which rely on echocardiographic data, are too often incorrect [6-12]. Cardiovascular magnetic resonance (CMR) with its tomographic approach to ventricular measurements, ability to quantify flow across vessels and valves, and capacity to identify late gadolinium enhancement (LGE) consistent with endocardial fibroelastosis (EFE) [13], has emerged as a potentially important tool in the preoperative assessment of this population. Therefore, we sought to identify CMR parameters associated with successful conversion from a univentricular to biventricular circulation.

## Methods

### Subjects

A retrospective database review of all patients undergoing CMR at Boston Children’s Hospital from January 2005 through July 2012 was conducted. Subjects were included if they had the following: 1) BHLHS or RDAVC, 2) no conotruncal abnormalities (e.g., L-loop transposition), 3) a univentricular circulation at the time of CMR followed by either surgical or catheter intervention to establish a biventricular circulation, 4) ventricular volumetric data measured at CMR, and 5) follow-up information available for ≥3 months after the intervention for survivors of biventricular conversion. BHLHS patients were defined as those with hypoplastic (z-score < −2) or obstructed left heart structures during their treatment course in whom there was uncertainty regarding ability to support a biventricular circulation. RDAVC patients were those with endocardial cushion defects with LV hypoplasia and/or more than 50% of the common atrioventricular valve related to the RV. Patient demographics, clinical history, echocardiography and CMR data, and surgical details were abstracted from the medical record. The Committee on Clinical Investigation at Boston Children’s Hospital approved this study.

Our institutional approach to patients with BHLHS takes into account multiple patient-specific anatomic and physiologic factors, as well as parent and healthcare provider preferences, and has evolved over time [14]. Moreover, the varied age at referral and prior surgical history impose some heterogeneity in management. In general, left heart structure growth may be promoted by increasing left heart blood flow at the time of a bidirectional Glenn shunt by including a Blalock-Taussig shunt or right ventricle-to-pulmonary artery conduit and/or atrial septal restriction. Mitral valvuloplasty and LV EFE resection are employed but typically not in the neonatal period. Surgical or catheter aortic valvuloplasty are performed at any time. Fetuses diagnosed with evolving hypoplastic left heart syndrome may undergo in utero balloon aortic valvuloplasty [15]. The decision whether to attempt a biventricular conversion procedure was at the discretion of each patient’s physicians who took clinical status, echocardiography, catheterization, and CMR data into consideration.

### CMR

CMR was performed on a 1.5 T scanner (Philips Achieva, Philips Medical Systems, Best, the Netherlands). All studies were performed under general anesthesia. A typical imaging protocol consisted of 1) cine steady-state free precession imaging in an axial plane, ventricular long- and short-axis planes, and aortic arch long-axis plane; 2) gadolinium-enhanced magnetic resonance angiogram; 3) phase velocity flow measurements in the native and neo-aortic roots, descending aorta, branch pulmonary arteries, pulmonary veins, atrioventricular valves, and superior and inferior vena cavae; and 4) LGE imaging in ventricular long- and short-axis planes 10–20 minutes after the administration of contrast.

Ventricular and flow volumes were calculated using commercially available software (QMass and QFlow, MEDIS Medical Imaging Systems, the Netherlands) as previously described [16,17]. In RDAVC patients, ventricular volumes were measured by extrapolating the plane of the septum to the base of the heart. EFE was defined as LGE of the endocardial wall of the LV and papillary muscles in a pattern not following a coronary distribution [13].

### Echocardiography

Echocardiograms were performed using standard imaging protocols [18-20]. LV volumes were calculated using the following formula: 5/6 * short-axis area * long-axis length [19,20]. To ensure consistency in the study, ventricular measurements as well as mitral and aortic valve dimensions were re-measured by a single observer.

### Statistics

Descriptive statistics are shown as median (range) or mean ± standard deviation. The associations between predictor and outcome variables were evaluated using the Mann–Whitney *U* test and the Fisher’s exact test. Receiver operating characteristic (ROC) curves were constructed for candidate variables to identify discrimination thresholds associated with the primary outcome — transplant-free survival with a biventricular circulation. ROC curves were compared using the approach described by Hanley et al. [21]. Transplant-free survival between patient groups was compared using Kaplan-Meier analysis. Echocardiographic and CMR measurements were compared using Spearman rank correlation and a Bland-Altman analysis of agreement. Results were considered statistically significant if p ≤ 0.05. Statistical calculations were performed using SPSS versions 18 and 21 (IBM Corporation, USA).

## Results

During the study period, 32 patients (22 with BHLHS and 10 with RDAVC) with a univentricular circulation met the inclusion criteria and underwent CMR prior to a biventricular conversion procedure. An additional 32 patients (31 with BHLHS and 1 with RDAVC) underwent attempts at left ventricular recruitment during the study period but were excluded because they did not undergo biventricular conversion during the study period. Furthermore, 10 patients (2 with BHLHS and 8 with RDAVC) were excluded because they did not undergo a CMR examination prior to biventricular conversion. Referral for CMR was at the clinical team’s discretion.

### Borderline hypoplastic left heart structures group

#### Clinical data

Demographic, clinical history, CMR, echocardiographic, and catheterization parameters for the BHLHS group (n = 22) are summarized in Table 1. Seventeen patients (77%) underwent a prior procedure designed to increase blood flow into the LV by providing an additional source of pulmonary blood flow (e.g.*,* Blalock-Taussig shunt in a patient with a bidirectional Glenn shunt), and/or by restricting the atrial septal defect to direct more of the pulmonary venous return into the LV. The median time between echocardiogram and CMR was 1 day (0–89), and between catheterization and CMR was 0 days (0–90). The median time between the CMR examination and the attempted biventricular conversion was 3 days (0–198).

**Table 1:** **Demographic and clinical data in the BHLHS group**

**Parameter**	**Value**
**N**	**22**
Initial anatomy	
Multiple left heart obstructions	19 (86%)
Critical aortic stenosis	3 (14%)
Female	7 (32%)
In utero aortic valvuloplasty	9 (40%)
Age at CMR (mo)	39 (0–82)
Body surface area (m^2^)	0.59 (0.22-0.72)
Circulation at the time of CMR	
Prostaglandin infusion	3 (13%)
Norwood stage 1 palliation	2 (9%)
Hybrid stage 1 palliation	1 (5%)
Bidirectional Glenn shunt	15 (68%)
Total cavopulmonary connection	1 (5%)
Prior procedure to increase left heart flow	17 (77%)
Dual sources of pulmonary blood flow	13
Restriction of the atrial septal defect	16
CMR parameters	
Heart rate (beats/min)	110 (89–150)
LV EDV (ml/m^2^)	57 (21–88)
LV ESV (ml/m^2^)	21 (10–49)
LV stroke volume (ml/m^2^)	31 (4–60)
LV ejection fraction (%)	61 (20–79)
RV EDV (ml/m^2^)	82 (52–132)
RV ESV (ml/m^2^)	39 (22–82)
RV stroke volume (ml/m^2^)	42 (17–65)
RV ejection fraction (%)	54 (27–66)
LV-to-RV stroke volume ratio	0.8 (0.06-1.7)
MV-to-TV inflow ratio	1.0 (0.2-3.0)
LGE pattern consistent with EFE	16 (73%)
LA pressure (mm Hg)	14 (5–16)
LV ED pressure (mm Hg)	12 (8–22)
Biventricular procedure CPB time (min)	149 (92–239)
Biventricular procedure LOS (days)	25 (7–156)

Values are given as median (range) or n (%).

*Abbreviations*: *CMR* cardiovascular magnetic resonance, *CPB* cardiopulmonary bypass, *ED* end-diastolic, *EDV* end-diastolic volume, *EFE* endocardial fibroelastosis, *ESV* end-systolic volume, *HLHS* hypoplastic left heart syndrome, *LA* left atrium, *LGE* late gadolinium enhancement, *LOS* length of stay, *LV* left ventricle, *MV* mitral valve, *RV* right ventricle, *TV* tricuspid valve.

#### Outcomes

Univentricular to biventricular conversion was attempted surgically in 20 patients (91%) and by catheter balloon dilation of the aortic valve in 2 (9%). Of the 22 patients, 6 (27%) failed the biventricular conversion procedure. Three of the failures occurred prior to discharge: 1 died in the hospital after a 5 month post-operative course with multiple surgical and catheter interventions, 1 underwent heart transplant 6 months after conversion, and 1 had an attempted balloon aortic valvuloplasty for establishment of biventricular circulation but LV function did not recover and he underwent a stage 1 palliation 4 days later. In addition, there were 3 late failures. One patient with a good early hemodynamic result died at home of unknown causes 2 months after conversion. A second patient had LV diastolic dysfunction and RV hypertension after conversion, and died of a respiratory illness 15 months later. A third patient developed severe pulmonary hypertension and pulmonary vein stenosis, requiring takedown to a stage 1 Norwood procedure 3 months after conversion. The 16 transplant-free biventricular survivors had a median follow-up time after the biventricular conversion procedure of 40 months (4–84). During this time, 6 of these patients underwent mitral valve replacement and 3 had aortic valve replacement. Among the 13 subjects with RV systolic pressure data available, 5 had pressure greater than one-half systemic pressure and 8 had pressure less than one-half systemic pressure but greater than 25 mm Hg.

#### Parameters associated with transplant-free biventricular circulation survival

**Figure 1:**
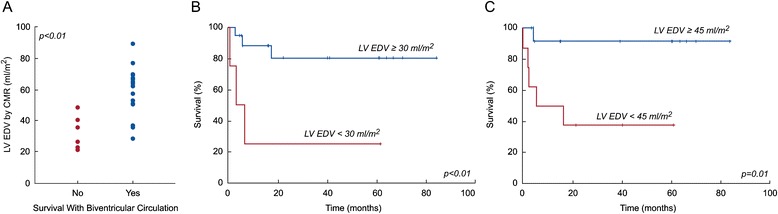
**Association of CMR-derived LV EDV and transplant-free, biventricular circulation survival at latest follow-up in BHLHS patients (n = 22). A)** LV EDV indexed to body surface area in those who did not (No) and who did (Yes) survive with a biventricular circulation; **B)** Kaplan-Meier plot of survival at follow-up with a biventricular circulation with LV EDV ≥30 ml/m^2^ versus <30 ml/m^2^; **C)** Kaplan-Meier plot of survival at follow-up with a biventricular circulation with LV EDV ≥45 ml/m^2^ versus <45 ml/m^2^.

**Figure 2:**
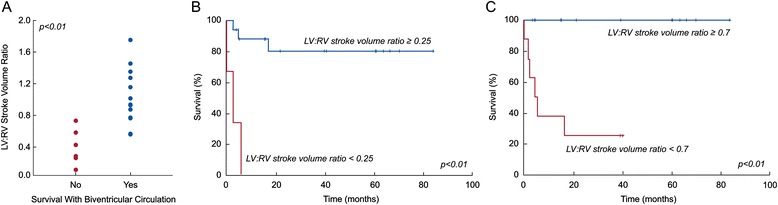
**Association of CMR-derived LV-to-RV stroke volume ratio and transplant-free, biventricular circulation survival at latest follow-up in BHLHS patients (n = 21). A)** LV-to-RV stroke volume ratio in those who did not (No) and who did (Yes) survive with a biventricular circulation; **B)** Kaplan-Meier plot of survival at follow-up with a biventricular circulation with LV-to-RV stroke volume ratio ≥ 0.25 versus < 0.25; **C)** Kaplan-Meier plot of survival at follow-up with a biventricular circulation with LV-to-RV stroke volume ratio ≥ 0.7 versus <0.7.

**Figure 3:**
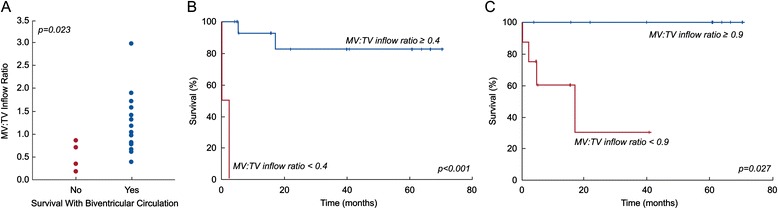
**Association of CMR-derived MV-to-TV inflow ratio and transplant-free, biventricular circulation survival at latest follow-up in BHLHS patients (n = 17). A)** MV-to-TV inflow ratio in those who did not (No) and who did (Yes) survive with a biventricular circulation; **B)** Kaplan-Meier plot of survival at follow-up with a biventricular circulation with MV-to-TV inflow ratio ≥ 0.4 versus < 0.4; **C)** Kaplan-Meier plot of survival at follow-up with a biventricular circulation with MV-to-TV inflow ratio ≥ 0.9 versus <0.9.

**Figure 4:**
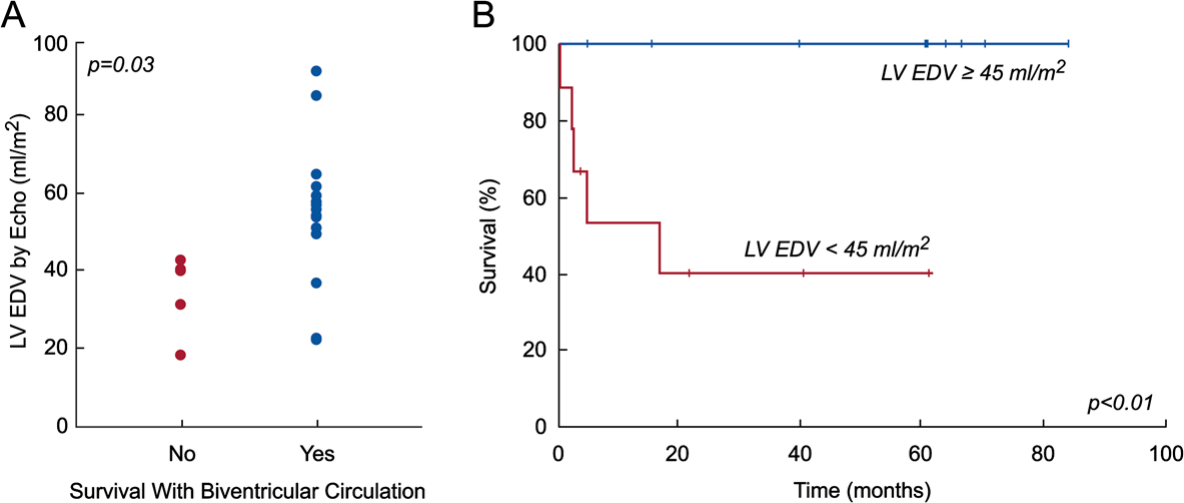
**Association of echocardiography-derived LV EDV and transplant-free, biventricular circulation survival at latest follow-up in BHLHS patients (n = 22). A)** LV EDV in those who did not (No) and who did (Yes) survive with a biventricular circulation; **B)** Kaplan-Meier plot of survival at follow-up with a biventricular circulation with LV EDV ≥ 45 ml/m^2^ versus < 45 ml/m^2^.

Table 2 compares patients who survived to latest follow-up with a transplant-free biventricular circulation to those who did not. Survivors of biventricular conversion were significantly older at the time of CMR and were more likely to have undergone a prior procedure to increase LV flow. They also had a significantly larger indexed CMR LV end-diastolic volume (EDV) (Figure 1A), a higher LV-to-RV stroke volume ratio (Figure 2A), and a higher mitral-to-tricuspid valve (MV-to-TV) inflow ratio (Figure 3A). A LGE pattern consistent with EFE was not associated with outcome, but 15 patients (68%) underwent EFE resection at some point in their treatment course, confounding interpretation of the significance of EFE. Based on echocardiography, survivors had a larger LV EDV (Figure 4A) and LV stroke volume. Mitral and aortic valve annulus diameter z-scores were similar between the groups. Left atrial and ventricular end-diastolic pressures at catheterization were also not significantly different between the groups.

**Table 2:** **Comparison between transplant-free survivors and non-survivors of biventricular conversion procedure in the BHLHS group**

	**Survivors**	**Non-survivors**	**P-value**
**N**	**16**	**6**	
Age at CMR (mo)	46 (0–82)	6.5 (0–46)	0.02
Age at conversion (mo)	47 (0–82)	5 (0–46)	0.02
Procedure to increase LV flow	14 (93)	2 (40)	0.01
CMR parameters			
LV EDV (ml/m^2^)	62 (28–88)	31 (21–48)	0.001
LV ESV (ml/m^2^)	23 (9–49)	15 (10–17)	0.02
LV stroke volume (ml/m^2^)	35 (20–60)	18 (4–32)	0.002
LV ejection fraction (%)	62 (45–79)	58 (20–67)	0.29
RV EDV (ml/m^2^)	76 (52–132)	97 (66–110)	0.07
RV ESV (ml/m^2^)	39 (22–82)	40 (31–46)	0.68
RV stroke volume (ml/m^2^)	38 (17–64)	59 (36–65)	0.02
RV ejection fraction (%)	51 (27–66)	58 (54–66)	0.08
LV-to-RV stroke volume ratio	0.9 (0.5–1.7)	0.3 (0.06–0.7)	<0.001
MV-to-TV inflow ratio	1.0 (0.4–2.0)	0.5 (0.2–0.9)	0.04
LGE pattern consistent with EFE	12 (80)	3 (60)	0.37
Echo parameters			
LV EDV (ml/m^2^)	57 (24–78)	31 (20–51)	0.02
LV stroke volume (ml/m^2^)	28 (12–37)	24 (12–27)	0.04
Mitral valve diameter z-score	-1.9 (-2.8 – -0.5)	-1.8 (-3.2 – -1.3)	0.55
Aortic valve diameter z-score	-2.1 (-3.9 – -1.4)	-1.8 (-2.1 – -1.4)	0.74
LA pressure (mm Hg)	13.5 (5–16)	14 (6–15)	1.0
LV ED pressure (mm Hg)	12 (8–22)	16.5 (12–22)	0.14
Biventricular conversion CPB time (min)	142 (92–260)	182 (122–239)	0.34
Biventricular conversion LOS (days)	19 (7–97)	87.5 (25–150)	0.20

Values are given as median (range) or n (%).

*Abbreviations*: *BHLHS* borderline hypoplastic left heart syndrome, *CMR* cardiovascular magnetic resonance, *CPB* cardiopulmonary bypass, *EDV* end-diastolic volume, *EFE* endocardial fibroelastosis, *ESV* end-systolic volume, *LA* left atrial pressure, *LGE* late gadolinium enhancement, *LOS* length of stay, *LV* left ventricle, *MV* mitral valve, *RV* right ventricle, *TV* tricuspid valve.

ROC analysis identified CMR and echocardiographic parameters and threshold values that best predicted survival with a transplant-free, biventricular circulation (Table 3). The area under the curve (AUC) for echocardiographic LV EDV did not differ significantly from that of CMR LV EDV (p = 0.15), CMR LV-to-RV stroke volume ratio (p = 0.09), or CMR MV-to-TV inflow ratio (p = 0.5). Using survival analysis to incorporate *time* to failure, these threshold values were all associated with significant differences in freedom from failure (Figures 1B and C, 2B and C, 3B and C, and 4B).

**Table 3:** **ROC Analysis for survival with a transplant-free, biventricular circulation**

**Parameter**	**AUC**	**P**	**Threshold value**	**Sensitivity**	**Specificity**
CMR LV EDV	0.93	0.006			
			30 ml/m^2^	94%	50%
			45 ml/m^2^	81%	83%
CMR LV-to-RV stroke volume ratio	0.96	0.009			
			0.25	100%	50%
			0.70	87%	100%
CMR MV-to-TV inflow ratio	0.89	0.02			
			0.40	100%	50%
			0.90	64%	100%
Echocardiographic LV EDV	0.83	0.03			
			45 ml/m^2^	75%	100%

*Abbreviations*: *AUC* area under the curve, *CMR* cardiovascular magnetic resonance, *EDV* end-diastolic volume, *LV* left ventricular, *MV* mitral valve, *RV* right ventricle, *TV* tricuspid valve.

The small sample size precluded multivariable analysis. However, data for all 4 threshold values (CMR-derived LV EDV ≥ 45 ml/m^2^, LV-to-RV stroke volume ratio ≥ 0.7, MV-to-TV inflow ratio ≥ 0.9, and echocardiography-derived LV EDV ≥ 45 ml/m^2^) were available for 19 patients. Among these, all 14 patients who met at least 2 of the 4 threshold values survived to follow-up with a biventricular circulation, compared to 1 of 5 patients who met only 0 or 1 of these 4 criteria (p = 0.001). This combination of 4 thresholds yielded a sensitivity of 93% with a specificity of 100%, compared to echocardiography-derived LV EDV ≥ 45 ml/m^2^ alone, which had a sensitivity of 75% and a specificity of 100%.

#### Comparison between echocardiography and CMR measurements

**Figure 5:**
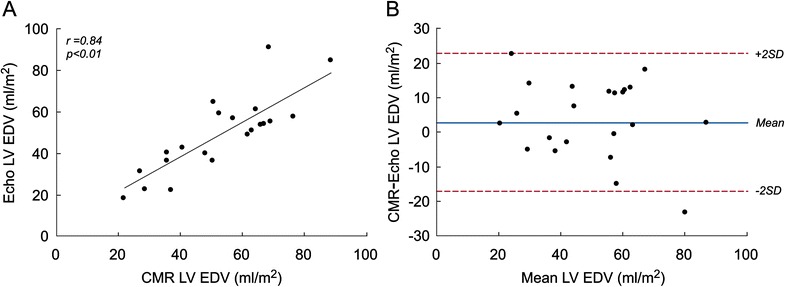
**Comparison between echocardiographic and CMR-derived indexed LV EDV in patients with BHLHS (n = 22). A)** Correlation plot. **B)** Bland-Altman agreement plot with a mean difference of 3.2 ± 10.5 ml/m^2^.

Echocardiography and CMR derived LV EDV measurements were closely correlated (r = 0.81, p < 0.001; Figure 5A) but had only fair agreement, with CMR values being larger (mean difference 3.2 ± 10.5 ml/m^2^; Figure 5B). Similarly, echocardiography and CMR derived LV ejection fraction measurements also correlated (r = 0.75, p < 0.001) but had only fair agreement, with CMR values being greater (mean difference 2.9 ± 8.0%).

### Right-dominant atrioventricular canal group

#### Clinical data

Demographic, clinical history, and CMR parameters for the RDAVC group (n = 10) are summarized in Table 4. Contemporaneous echocardiograms were available in all patients and catheterizations in 9 patients. The median time between echocardiogram and CMR examination was 0 days (0–8), and between catheterization and CMR examination was 0 days (0–90). The median time between the CMR examination and the attempted biventricular conversion was 2.5 days (0–22).

**Table 4:** **Demographic and clinical data in the RDCAVC group**

**Parameter**	**Value**
N	10
Female	4 (40%)
Trisomy 21	6 (60%)
Age at CMR (mo)	6 (2–75)
Body surface area (m^2^)	0.34 (0.26-0.65)
Circulation at CMR	
Pulmonary artery band	3 (30%)
Norwood stage 1 palliation	2 (20%)
Bidirectional Glenn shunt	3 (30%)
Bidirectional Glenn + pulmonary artery band	2 (20%)
CMR parameters	
LV EDV (ml/m^2^)	32 (22–39)
LV ESV (ml/m^2^)	13 (8–18)
LV stroke volume (ml/m^2^)	18 (13–23)
LV ejection fraction (%)	58 (51–67)
RV EDV (ml/m^2^)	137 (58–171)
RV ESV (ml/m^2^)	61 (22–94)
RV stroke volume (ml/m^2^)	68 (36–90)
RV ejection fraction (%)	54 (45–67)
LV-to-RV stroke volume ratio	0.27 (0.19-0.44)
LA pressure (mm Hg)	8 (7–9)
LV ED pressure (mm Hg)	9 (7–12)
Biventricular procedure CPB time (min)	172 (106–250)
Biventricular procedure LOS (days)	33 (11–253)

Values are given as median (range) or n (%).

*Abbreviations*: *CMR* cardiovascular magnetic resonance, *CPB* cardiopulmonary bypass, *EDV* end-diastolic volume, *ESV* end-systolic volume, *BHLHS* borderline hypoplastic left heart syndrome, *LA* left atrium, *LOS* length of stay, *LV* left ventricle, *MV* mitral valve, *RV* right ventricle, *TV* tricuspid valve.

#### Outcomes

All patients survived the biventricular conversion procedure and had a biventricular circulation at the time of hospital discharge. Over a median post-conversion follow-up period of 28 months (3–51), 1 patient with recurrent mitral stenosis and LV outflow tract obstruction died at home from a respiratory illness 12 months after conversion. Among the survivors, 1 patient required mitral valve replacement and none required aortic valve replacement. No patients had RV systolic pressure greater than one-half systemic pressure, 8 were less than one-half systemic but greater than 25 mm Hg, and 1 was less than 25 mm Hg. The small sample size precluded an analysis of the parameters associated with a transplant-free biventricular circulation. Successful biventricular conversions were seen in patients with an LV EDV as low as 22 ml/m^2^ and a LV-to-RV stroke volume ratio as low as 0.19.

#### Comparison between echocardiography and CMR measurements

**Figure 6:**
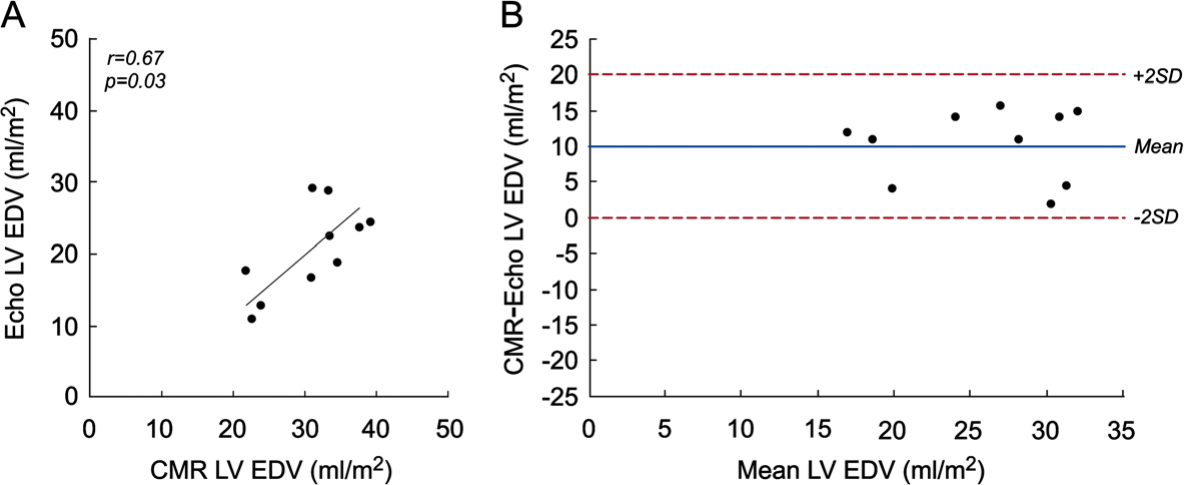
**Comparison between echocardiographic and CMR-derived indexed LV EDV in patients with RDAVC (n = 10). A)** Correlation plot. **B)** Bland-Altman agreement plot with a mean difference of 10.3 ± 5.0 ml/m^2^.

Echocardiography and CMR derived LV EDV had modest correlation (r = 0.67, p = 0.03; Figure 6A) and only fair agreement, with CMR values being larger (mean difference 10.3 ± 5.0 ml/m^2^; Figure 6B). Echocardiography and CMR derived LV ejection fraction measurements were not significantly correlated (r = −0.44, p = 0.21) and showed poor agreement, with higher ejection fraction by CMR compared to echocardiography (mean difference 14.3 ± 15.0%).

## Discussion

This study is the first to report CMR findings predictive of a successful conversion from univentricular to biventricular circulation in patients with left heart hypoplasia. We found that a greater LV EDV, LV-to-RV stroke volume ratio, and MV-to-TV inflow ratio were associated with a higher rate of survival with a biventricular circulation in patients with BHLHS. We were also able to identify threshold values for these parameters that were associated with successful biventricular conversion. These data will facilitate interpretation of CMR measurements in patients with BHLHS being considered for biventricular conversion and assist with risk stratification. The high success rate for biventricular conversion in the RDAVC patient group precluded identification of predictor parameters. Nevertheless, our data provide new information on the range of CMR-derived ventricular parameters in RDAVC that are compatible with biventricular conversion.

Grosse-Wortmann et al. previously demonstrated the feasibility of CMR in a cohort of 20 consecutive neonates with left heart hypoplasia [22]. However, because the number of patients in their study who underwent biventricular repair was small (n = 16) and only 1 patient did not survive a biventricular management strategy, they were unable to assess for factors associated with successful biventricular repair. Our study was not restricted to neonates and many patients had undergone prior interventions to increase blood flow through the LV with the aim of promoting a biventricular repair [14]. Therefore, it may assist with risk stratification not only for neonates with BHLHS who are being considered for biventricular conversion, but also for older patients who have undergone previous univentricular palliation or LV recruitment maneuvers.

The factors we identified as predicting a higher likelihood of successful biventricular conversion – larger LV EDV, LV-to-RV stroke volume ratio, and MV-to-TV inflow ratio are inter-related and plausible from a physiologic standpoint. It is not surprising that a larger LV that is supporting a greater proportion of the cardiac output is more likely to sustain a full cardiac output at the time of biventricular conversion. Moreover, ventricles that are larger may have greater compliance allowing ventricular filling at physiologically acceptable pressures, which is often a limiting factor in this patient population. Similarly, a mitral valve that can accommodate greater inflow should be more likely to tolerate a complete cardiac input at the time of biventricular conversion. These factors are also consistent with a prior publication from our institution that found that degree of atrial septal restriction was associated with outcome [14] as it is likely that greater restriction results in more mitral inflow (and a higher MV-to-TV ratio), a larger LV EDV, and potentially a higher LV-to-RV stroke volume ratio.

### Comparison of CMR and echocardiography measurements

We found good correlation between CMR and echocardiography for LV volumes and ejection fraction in the BHLHS group, and to a lesser extent for LV volumes in the RDAVC group. There was not a significant correlation for LV ejection fraction in the RDAVC group. However, despite the good correlations between modalities in the BHLHS group, the limits of agreement were relatively wide, which indicates that substantial differences in measurements beyond a systematic bias are present. Small chamber size, abnormal chamber geometry related to LV compression by the dilated RV, and abnormal papillary muscle architecture may contribute to differences between modalities. The impact of these confounders is likely more pronounced in patients with RDAVC, explaining the poorer correlations between echocardiography and CMR in this group.

In contrast to our study, Grosse-Wortmann et al., found poor correlation between measurements of ventricular volume and function between the two modalities even for the LV [22]. Their study used the monoplane Simpson’s method for calculating LV volumes by echocardiography, rather than the 5/6 * area * length method used in ours. Moreover, they analyzed patients with and without an atrioventricular canal defect together whereas we analyzed the two lesions separately.

### Utility of CMR

Although several studies have identified echocardiographic parameters to predict successful biventricular conversion in both BHLHS [12,23-25] and RDAVC [26-29], decision-making remains challenging. In a multicenter, retrospective evaluation of 362 neonates with critical left ventricular outflow obstruction, Hickey et al. found that inappropriate pursuit of biventricular repair in patients with borderline left heart structures was common and resulted in poor long-term survival compared to that predicted with single ventricle palliation [6]. Similarly, in a retrospective study of 45 patients with RVDAVC from a single institution, Szwast et al. found that 18% of patients who underwent biventricular repair did not survive to follow-up [26]. These suboptimal results utilizing echocardiography led us to investigate the utility of CMR-derived parameters for decisions regarding biventricular repair.

Although we identified 3 CMR parameters that were associated with biventricular survival in the BHLHS group, the individual AUCs for these were not significantly different from that of LV EDV by echocardiography. However, since no single parameter is a perfect predictor, the use of several parameters, some based on ventricular volume and others on flow data, may result in better risk stratification. In our study, the small sample size precluded multivariable analysis. However, using a combination of 4 thresholds, including CMR parameters, improved sensitivity compared to a single echocardiographic threshold, thereby highlighting the potential additive value of CMR. Furthermore, CMR can provide information regarding the presence and distribution of LGE when EFE resection is being considered.

In patients with RDAVC, the small number of patients in our cohort and favorable outcomes precluded discriminant analysis. Nevertheless, it is worth noting that successful biventricular conversions were seen in patients with a LV EDV as low as 22 ml/m^2^ and a LV-to-RV stroke volume ratios as low as 0.19. As more patients with RDAVC undergo CMR and biventricular conversion, predictive threshold values for LV EDV will likely emerge. Given the poor correlation and agreement between CMR and echocardiographic measurements of LV EDV, CMR-derived values may prove to be more powerful. This information is particularly important for managing patients who are at increased risk with a univentricular palliation, such as those with trisomy 21 or with elevated pulmonary artery pressure.

### Limitations

Our study has a relatively small sample size as BHLHS and RDAVC cases are rare. Moreover, there is a potential selection bias because not all patients at our institution were referred for CMR prior to a univentricular to biventricular conversion procedure. Our study design also excluded patients who underwent CMR but did not undergo a biventricular conversion, perhaps based on the CMR data itself. All of these factors could have potentially decreased the power of the study to identify CMR parameters associated with successful biventricular conversion. Nevertheless, we were able to identify 3 parameters strongly associated with outcome. One of these parameters, the MV-to-TV ratio, utilizes phase contrast measurements of atrioventricular valve blood inflow. The accuracy of these measurements may be limited by the through-plane motion of the valves relative to the fixed imaging plane [30]. However, this error is at least partially mitigated in our study by the use of the ratio of atrioventricular valve flow as both valves move in the same direction. Several studies have identified the presence of extensive EFE as a risk factor for unsuccessful biventricular repair [24,31,32]. In our study, the high rate of EFE resection confounded the interpretation of EFE data. Finally, this study was not designed to evaluate the long-term outcome of a biventricular repair in these patients; it is possible that with longer follow-up additional biventricular patients will undergo heart transplantation, takedown to a univentricular circulation, or death.

## Conclusions

This study provides novel data on CMR parameters predictive of successful conversion from univentricular to biventricular circulation in patients with left heart hypoplasia. Greater CMR LV EDV, LV-to-RV stroke volume ratio, and MV-to-TV inflow ratio were associated with a higher rate of survival with a biventricular circulation in patients with BHLHS. The addition of CMR parameters to echocardiographic measurements improved the sensitivity for predicting successful conversion. Successful biventricular conversions were seen in RDAVC patients with LV EDV as low as 22 ml/m^2^ and LV-to-RV stroke volume ratios as low as 0.19. More studies are needed in this difficult patient group to further refine risk stratification and improve patient management.

## Abbreviations

AUC: Area under the curve
BHLHS: Borderline hypoplastic left heart structures
CMR: Cardiovascular magnetic resonance
EDV: End-diastolic volume
EFE: Endocardial fibroelastosis
LGE: Late gadolinium enhancement
LV: Left ventricular
MV: Mitral valve
RDAVC: Right-dominant atrioventricular canal
ROC: Receiver operating characteristic
RV: Right ventricular
TV: Tricuspid valve
